# MXene-Based Nucleic Acid Biosensors for Agricultural and Food Systems

**DOI:** 10.3390/bios12110982

**Published:** 2022-11-07

**Authors:** Weizheng Wang, Sundaram Gunasekaran

**Affiliations:** Department of Biological Systems Engineering, University of Wisconsin-Madison, 460 Henry Mall, Madison, WI 53706, USA

**Keywords:** MXene, nucleic acid, biosensors, agricultural and food system, contaminants

## Abstract

MXene is a two-dimensional (2D) nanomaterial that exhibits several superior properties suitable for fabricating biosensors. Likewise, the nucleic acid (NA) in oligomerization forms possesses highly specific biorecognition ability and other features amenable to biosensing. Hence the combined use of MXene and NA is becoming increasingly common in biosensor design and development. In this review, MXene- and NA-based biosensors are discussed in terms of their sensing mechanisms and fabrication details. MXenes are introduced from their definition and synthesis process to their characterization followed by their use in NA-mediated biosensor fabrication. The emphasis is placed on the detection of various targets relevant to agricultural and food systems, including microbial pathogens, chemical toxicants, heavy metals, organic pollutants, etc. Finally, current challenges and future perspectives are presented with an eye toward the development of advanced biosensors with improved detection performance.

## 1. Introduction

The detection technologies for quality and safety monitoring in agricultural and food systems help improve our overall quality of life. Conventional methods to detect biological and/or chemical entities include plate-counting tests, enzyme-linked immunosorbent assays (ELISA), liquid and gas chromatography (LC and GC), etc. These methods are slow and expensive, requiring complicated pre-treatment of samples, sophisticated instruments, and highly qualified personnel to perform the tests inside a laboratory. To this end, biosensors are being developed to afford inexpensive, portable, and easy-to-operate devices for on-field detection. Biosensors are usually composed of two functional elements: (1) a bio-recognizer (e.g., enzyme, antibody, or nucleic acid (NA) sequences) that specifically recognizes and/or conjugates with target molecules and (2) a physiochemical transducer that translates the biorecognition event into a corresponding detectable signal [1,2].

NAs in oligomerization forms are usually integrated into biosensors as biorecognition elements due to their high specificity toward different target entities [3]. The most well-known NAs are deoxyribonucleic acid (DNA) and ribonucleic acid (RNA). According to the development stage, there are three kinds of NA biosensors, each with a unique function: (1) genosensor, (2) aptasensor, and (3) DNAzyme/aptazyme biosensors [4,5]. These biosensors are now widely explored and used in different areas, e.g., food safety monitoring, environment analysis, etc. [6].

The performance of NA biosensors is significantly increased by incorporating nanomaterials (NMs) that are employed as transducers, which further facilitate the immobilization of NA. NMs can be modified by different ligands that enhance NA–ligand interactions in terms of their superb physicochemical properties. Additionally, NMs with exceptional optical, electrical, and thermal conductivities and mechanical performance help establish various types of sensing devices with high sensing performance. A high surface-area-to-volume ratio of NMs provides a larger surface area for more surface functionalization and consequently increases the NA-loading efficiency and surface immobilization, which is critical to improving the detection sensitivity [7].

**Figure 1:**
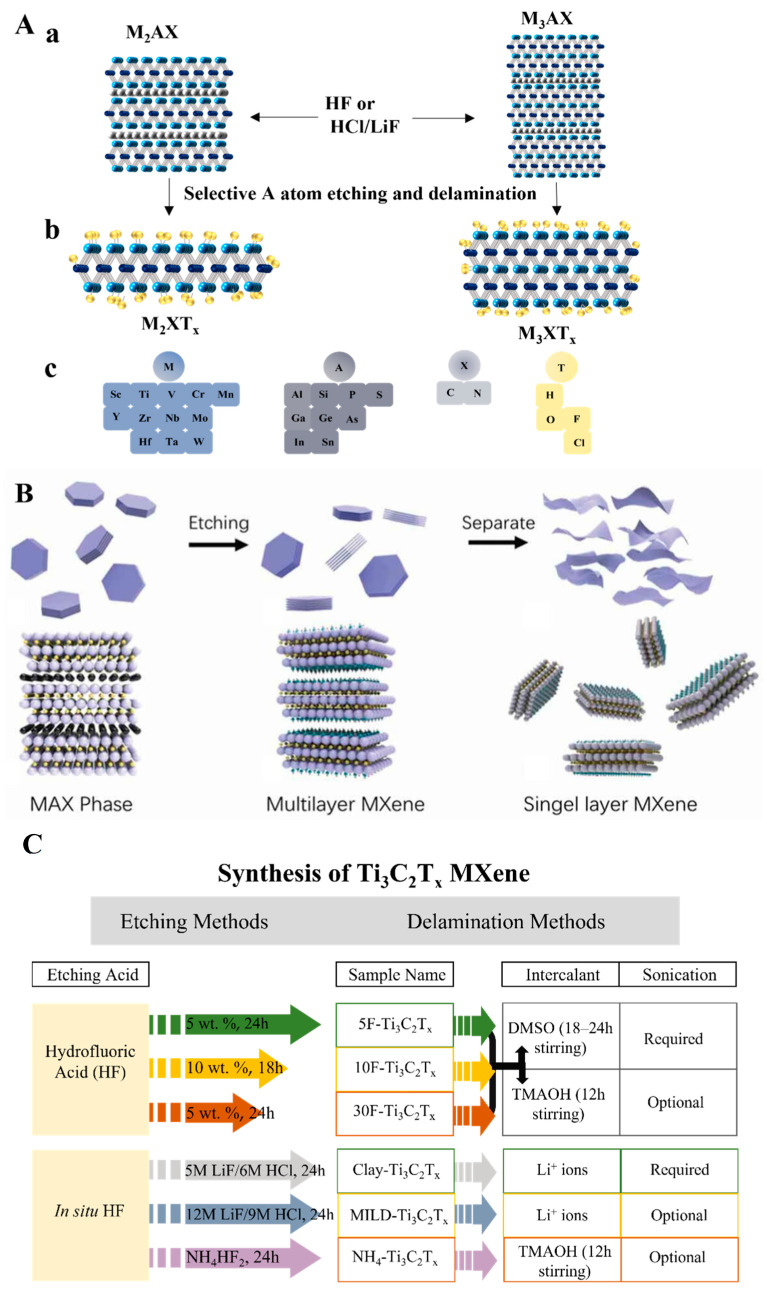
(**A**) MAX phase and its etched products. (**a**) Three typical MAX phase structures with selective etching sites (atoms in red). (**b**) Selectively etched products (MXene) with surface modification (atoms in yellow). (**c**) Atoms in MAX phases and MXene structures. Redrawn based on Ref. [11]. (**B**) Illustration for MXene top-down synthesized process from its precursor. Reproduced from Ref. [18]. (**C**) General idea for MXene top-down etched from MAX phases with two typical routes. Redrawn with permission from Ref. [21].

Two-dimensional (2D) NMs with sheet-like architectures, which due to their large surface area provide abundant reactive sites for ligand modification to bind with NA, are commonly used in biosensors [8]. Among these are 2D graphene-like MXenes that are hexagonal layered NMs such as carbides, nitrides, and carbonitrides and contain early transition metals [9]. They are usually synthesized from their parent bulk ceramic called the MAX phase, notated as M_n+1_AX_n_, where M is an early transition metal, e.g., Ti, Mo, Nb, etc.; A is an *sp* element that is primarily in group 13 or 14 from the periodic table; X is a carbon or nitrogen element (Figure 1(Aa,Ac)) [10]. After the *sp* element is etched, the remaining structures of the MAX phase are collectively called MXene (Figure 1(Ab,Ac)) [11]. Depending on the chemical etching method, various additional elements or groups may be anchored on the surface of MXene via hydrogen bonds or van der Waals bonds. The final MXene is represented as M_n+1_X_n_T_x_, where T represents different modifiers on the surface (typically fluoride (-F), chloride (-Cl), hydroxyl (-OH), oxygen (=O), etc.) [12,13]. Several unique merits in final MXene products make MXene a better alternative 2D NM in replacing other current 2D NMs. Based on the way they are synthesized, MXenes exhibit high reductivity that can reduce oxidizing reagents and even metal salt precursors without adding extra reducing agents [14]. Due to abundant M and X components, MXenes have a high electrical conductivity (6000–8000 S cm^−1^), which is highly beneficial to electron (e^−^) movement throughout the materials [15]. Additionally, the large surface area and the extra chemical terminals of MXene provide a platform for binding bio-recognition elements and modifying the surface that enhances biosensing performance [16]. Notably, with different extra atoms/polar groups terminating, the surface of the MXene products is much more easier to be chemically modified, consequently further facilitating and stabilizing the NA immobilization.

Herein we discuss and summarize the research efforts in developing NA biosensors based on 2D MXene for quality and safety monitoring of agri-food systems. The major classes of NA biosensors, such as genosensors, aptasensors, and DNAzyme sensors, are examined emphasizing their sensing mechanisms and fabrication details. We also present the future challenges and opportunities for NA biosensors in the monitoring of agri-food systems.

## 2. MXene Synthesis and Characterization

There are over 25 top-down and bottom-up approaches for the synthesis of MXene [17]; the top-down approaches are more widely applied (Figure 1B) [18]. As mentioned, MXenes are usually prepared from their MAX phase precursors, where the atomic layer A is removed by chemical or mechanical methods. Owing to covalent, partial ionic, and metallic bonds, the hexagonal layered MAX phase is observed in a stacked form and shows weakly metallic properties [19]. Unlike stacked graphite with π–π bonds, the bulk MAX phase is usually tightly held by partial ionic bonds, making it hard for MXene to separate from its precursors by using mechanical methods such as ultrasonication or mechanical exfoliation alone [20]. Therefore, selective chemical exfoliation is the predominant way to remove the atomic interlayer. The exfoliation is typically done by either hydrofluoric (HF) acid etching or in situ HF etching method (Figure 1C) [21].

Chemical vapor deposition (CVD) is one of the most common ways to scalably and controllably produce 2D NMs in the bottom-up method, where layered high-quality 2D NMs are grown on a substrate at a reasonable cost [22]. Consequently, top-down and bottom-up approaches are extensively explored to develop various MXene composites and structures for NA biosensor applications. This section introduces several popular MXene preparation methods.

### 2.1. Top-Down Etching of MAX Phase Precursors

#### 2.1.1. HF Etching

**Figure 2:**
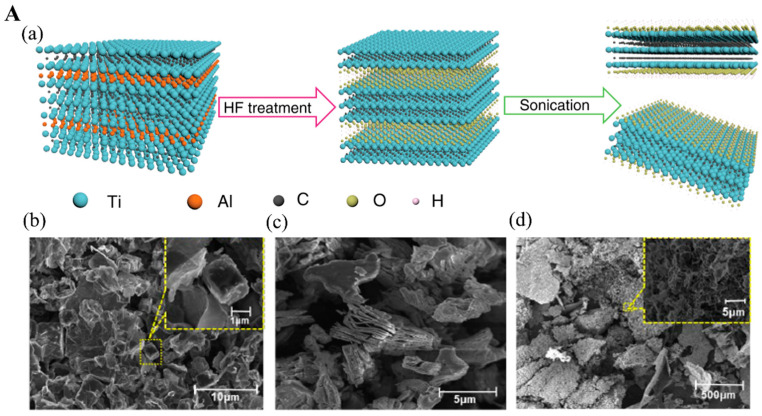

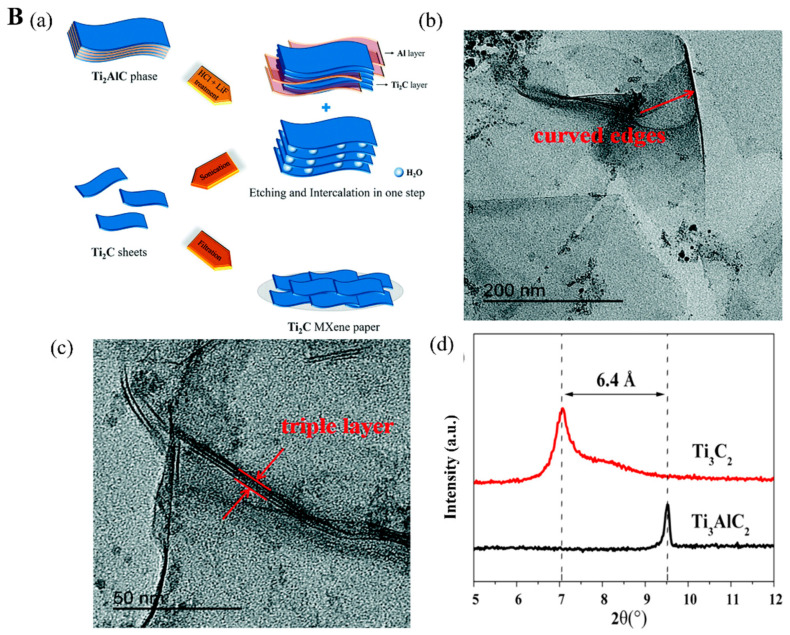
(**A**). (**a**) Schematic of Ti_3_C_2_T_x_ MXene synthesis via HF-etching. The SEM images for (**b**) Ti_3_AlC_2_ MAX phase, (**c**) multilayer Ti_3_C_2_T_x_ MXene, (**d**) delaminated Ti_3_C_2_T_x_ MXene nanosheets. Reproduced with permission from Refs. [24,28]. (**B**). (**a**) Ti_2_C MXene synthesis process. (**b**,**c**) TEM for exfoliated Ti_2_C MXene nanosheets. Reproduced with permission from Ref. [30]. (**d**) X-ray diffraction spectra for Ti_3_C_2_ MXene its MAX phase precursor. Reproduced with permission from Ref. [31].

HF etching can selectively etch the A interlayer without damaging other adjacent atomic structures [23]. Typically, concentrated aqueous HF solution (50%) is mixed with MAX phase powder at room temperature for several hours (Figure 2(Aa)) [24]. During this, the A layer is selectively removed by the strong acid solution, releasing hydrogen gas (H_2_) [25]. Concomitantly, other functional groups including -F, -OH, and =O are present on the surface of the remaining structure by van der Waals bonds [26]. The resulting solution is further centrifuged to precipitate the powder products, followed by washing with deionized (DI) water several times to remove excess HF solution. To maximize the abilities of MXene, it is critical to collect the separated MXene nanosheets using different intercalants where the interlayer spacing between the nanosheets is expanded enough to diminish the interaction between each adjacent nanosheet [27]. Dimethyl sulfoxide (DMSO) is usually used as an intercalant solvent that assists in delamination, followed by sonication. Other intercalating compounds, especially tetraalkylammonium salts such as tetrabutylammonium hydroxide (TBAOH), can also be used to separate MXene nanosheets with the aid of sonication and eventually increase d-spacing to achieve thermodynamic stability [27].

The scanning electron microscopy (SEM) pictures of the typical titanium-based MAX phase (Ti_3_Al_1.15_C_2_ powder) with its etched product (Ti_3_C_2_ MXene) nanostructures obtained before and after HF etching are shown in Figure 2(Ab,Ac). Figure 2(Ac) shows an accordion-like 2D Ti_3_C_2_ nanosheet structure differentiated from Figure 2(Ab). After TBAOH delamination, the exfoliated Ti_3_C_2_ nanosheets exhibit several nanobridges at the outer edges, demonstrating that the Ti_3_C_2_ nanosheets are successfully exfoliated from their MAX phase precursors (Figure 2(Ad)) [28]. This pristine MXene synthesized by the HF method possesses excellent electrochemical, optical, chemical, and mechanical properties [29].

#### 2.1.2. In Situ HF Etching

Unlike in the HF etching method where pure aqueous HF solution is added, in the in situ HF method, a mixture of molten fluoride or bifluoride-based salts and hydrogen chloride (HCl) is used as etchants. During the etching process, fluoride ion (F^−^)-containing salts including lithium fluoride (LiF) and ammonium hydrogen difluoride (NH_4_HF_2_) are added into the MAX phase to remove the A atomic interlayer resulting from the reactions between F^-^ and A layer [32]. Solvated lithium ions (Li^+^) intercalated between the interlayer of the MAX phase can also drive the delamination process, ultimately producing exfoliated MXene nanosheets [33]. This method offers several advantages over the HF etching method, such as the release of a low amount of hazardous compounds, mild conditions, and simultaneous delamination as a result of cations (e.g., Li^+^) [34].

Li et al. [30] fabricated Ti_2_CT_x_ MXene nanoflake-based paper using the aqueous HCl and LiF salts for etching the Al interlayer of the Ti_2_AlC precursor (Figure 2(Ba)). Individual exfoliated Ti_2_CT_x_ MXene nanoflakes were obtained after the mild sonication of the resultant products due to the intercalation of solvated Li^+^ between the exfoliated layers. Finally, individual Ti_2_CT_x_ MXene nanoflakes were dispersed with carbon nanotubes to prepare the Ti_2_CT_x_ MXene paper. The transmission electron microscope (TEM) (Figure 2(Bb,Bc)) images show a majority of LiF/HCl-Ti_2_CT_x_ MXene with wrinkled and curved edges, which is attributed to the high flexibility of the MXene paper. In another investigation, an aqueous NH_4_HF_2_ solution is mixed with MAX phase (Ti_3_AlC_2_), which functions as an etchant solution to remove the Al layer and produce the Ti_3_C_2_T_x_ MXene nanosheets [31]. Typically, MAX phases are highly anisotropic and their *c-lattice* parameter can easily be measured by powder X-ray diffraction (XRD) based on a (002) peak at 2θ around 10° [35,36]. According to Figure 2(Bd), the exfoliated Ti_3_C_2_ nanosheets show the (002) peak at 2θ = 7.1° shifted from 2θ = 9.6° (for Ti_3_AlC_2_ precursor) with calculated *c-lattice* parameter reaching 24.9 Å based on Bragg equation due to NH_4_^+^ intercalation effect. It indicates that the *d*-spacing increases while the thickness of Ti_3_C_2_ nanosheets reduces [13,31]. The NH_4_HF_2_ etching method provides the resulting Ti_3_C_2_ nanosheets with large interplanar spacing comparable to other fluoride-based etched methods, which allows the Ti_3_C_2_ nanosheets to be obtained in a single-step process. Ti_3_C_2_T_x_ MXene with fewer layers has higher conductivity (4600 ± 1100 S cm^−1^) with excellent field-effect electron mobility of 2.6 ± 0.7 cm^2^ V^−1^ S^−1^, which facilitates the electron transferring throughout the transducer, finally enhancing the sensitivity of electro-related biosensors [37]. Additionally, ultrathin MXene micropatterned on glass substrates for field-effect transistor fabrication provides a highly sensitive sensing platform for dopamine neurotransmitters analysis [38].

### 2.2. Bottom-up Synthesis of MXene

**Figure 3:**
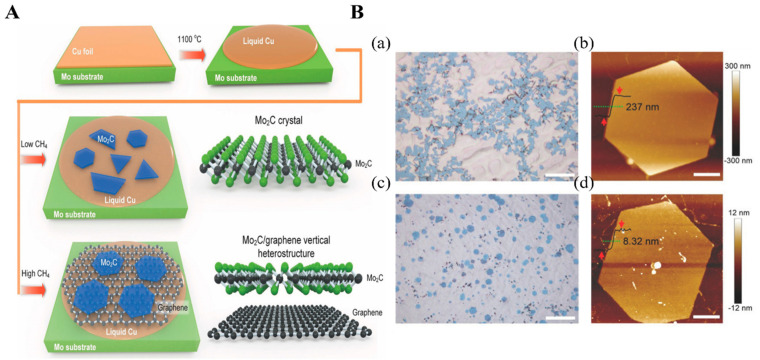
(**A**) Illustrative diagram for the Mo_2_C products growth under the high and low flow rates of CH_4_ gas. (**B**) Surface morphology of synthesis crystals: (**a**,**c**) Mo_2_C crystals’ physical distribution on Cu surface and graphene under optical images under low (**a**) and high (**c**) CH_4_ flow rates; (**b**,**d**) topological image of hexagonal Mo_2_C structures on the Cu surface, (**b**,**d**) graphene surface. Reproduced with permission from Ref. [42].

CVD is one of the most common bottom-up methods for high-quality and large-area NMs fabrication that is widely used in multiple practical applications, including optoelectronics and solar cell devices [39,40]. Typically, the precursors and substrate are preplaced in a chamber where extremely high temperatures are applied to heat or decompose the precursors, leading to the preheated or decomposed precursors growing on the surface of the substrate [41]. Consequently, more purified NM structures are developed. Several 2D MXenes were achieved via CVD. For example, ultrathin 2D molybdenum carbide (Mo_2_C) MXene was collected from the graphene-templated growth of Mo_2_C MXene film by the CVD method (Figure 3A) [42]. In the CVD process, copper (Cu) covered on the Mo foil was placed in a quartz chamber tube where 1100 °C was applied under a continuous flow of gaseous hydrogen (H_2_) and carbon-source methane (CH_4_). As a result, the morphology of the final Mo_2_C crystal MXene is largely dependent on the CH_4_ flow rate. With a low rate of CH_4_ flow, homogeneous structures of Mo_2_C crystal are obtained, while with a high rate of CH_4_ flow Mo_2_C/graphene heterostructures are obtained (see Figure 3(Ba,Bc)). When prepared on a Cu surface under a high CH_4_ flow rate, the final Mo_2_C crystal MXene had a more uniform structure with significantly lower thickness than when prepared on a graphene surface (Figure 3(Bb,Bd)). This indicates that CVD-synthesized graphene drastically suppresses the growth rate, thus reducing the thickness of as-prepared Mo_2_C MXene. Though CVD can control the thickness of the MXene nanosheets, a slow synthesis with a lower yield limits its use in MXene preparation.

Several synthetic methods discussed in this section have been proposed and successfully used to prepare different types of MXene and manipulate their surface, which largely influences the optical/fluorescent and electrochemical properties of the final MXene products. These altered properties further extend the use of MXenes for biosensing applications.

## 3. MXene in NA Biosensors

The NA sequences are used as biorecognition elements in biosensors to provide target-specific information to the transducer where the biorecognition event is transformed into a measurable signal such as optical, electrochemical, etc. While the specificity of the target depends on the biorecognition element, the sensitivity of the detection signal depends on the properties of the transducer element, for example, its electrical conductivity. Typically, 2D NMs, such as MXene, graphene, etc., that possess a large surface area to volume ratio can provide high signal sensitivity.

Single-strand DNA (ssDNA) or RNA macromolecules naturally hybridize with their complementary strands to form double-stranded molecules with high stability. Thus, NA-based bio-probes are widely used in different biosensors as biorecognition elements [43]. The hybridization performance benefits the genoanalytical device development for NA analysis and monitoring in many areas, particularly in DNA microarray, gene lab-on-a-chip technology, and amplification process in a polymerase chain reaction (PCR) [44,45]. The considerable evolution of NA fragments on high affinity and specificity to non-NAs proceeded when those specific fragments were isolated from large libraries incorporated with the systematic evolution of ligands by the exponential enrichment (SELEX) method [46,47]. Those NA fragments or aptamers have boosted applications of NA-based biosensors which are known as aptasensors [48]. NAs have also been shown to behave as a catalyst since Kruger et al. reported the first ribozymes [49]. Now DNAzymes, RNAzymes, aptazymes, and ribozymes play vital roles in biosensing systems.

NA-based biosensors can be divided into three categories based on their different abilities as bio-recognized probes: (1) genosensor, (2) aptasensor, and (3) DNAzyme/RNAzyme biosensor. An overview of these biosensors, their fabrication details, and the role of MXene in them are presented herein.

### 3.1. NA-Based Biosensor

#### 3.1.1. Genosensor

A genosensor is developed by taking advantage of the hybridization reaction between selected ssDNA or RNA with their complementary counterpart [50]. In this selected ssDNA or RNA, serving as the biorecognition element is immobilized on the transducer surface, which recognizes and hybridizes with the target DNA or RNA. The hybridization event is transmitted through a transducer and transformed into a measurable signal. While the specificity of the genosensor depends on the selection of the biorecognition element, the sensitivity of the genosensor is largely influenced by the structures and properties of the NMs that constitute the transducer.

**Figure 4:**
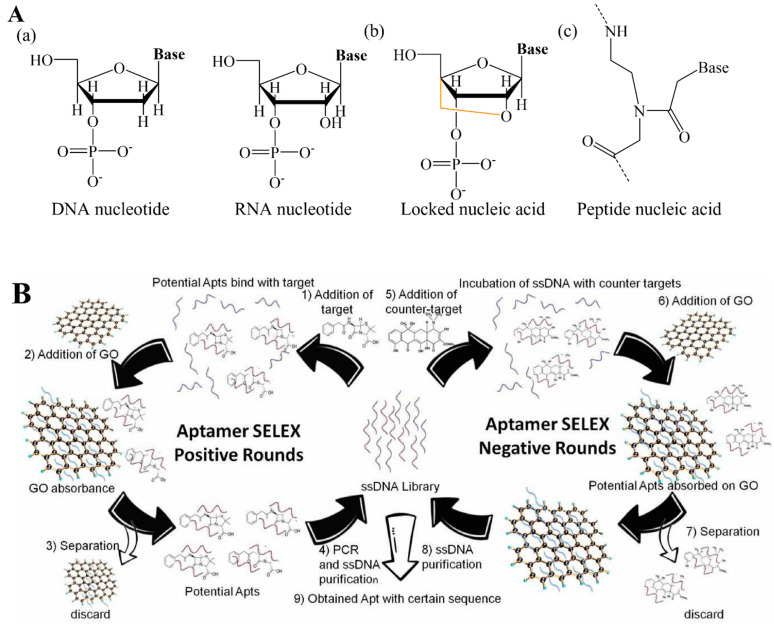
(**A**) Chemical structures of nucleic acids: (**a**) DNA and RNA nucleotide, (**b**) locked nucleic acid, (**c**) peptide nucleic acid. (**B**) Schematic illustration of aptamer selection by positive and negative SELEX methods. Reproduced with permission from Ref. [60]. Copyright 2022, Elsevier.

Several gene probes in natural or chemical-changed forms have been designed and utilized in genosensors [51,52]. Most commonly, natural nucleonic acid (NNA: DNA/RNA nucleotide) (Figure 4(Aa)) contributes to the major backbone of linear oligonucleotides, either pre- or in situ synthesis [53,54]. Nevertheless, hairpin-like oligonucleotides have been popularly applied recently. A hairpin oligonucleotide has a unique structure with a self-complementary characteristic that is patterned as a closed-stem zone, where base pairs are formed intramolecularly and an opened-loop zone exposed to the external environment with a capture sequence [55,56]. Chemically modified natural DNA/RNA analogs have emerged as promising critical components of the gene probes used in various genosensors. For example, one of the most popular analogs is locked nucleic acid (LNA), also known as bridged nucleic acid (BNA), which possesses superior RNA-binding affinity and excessively stable structure [57]. LNA is a bicyclic-based structure where 2’ oxygen and 4’ carbon are locked via an extra bridge in a flexible ribose motif (Figure 4(Ab)) [58]. With these exceptional properties, LNA is the most used in clinical diagnosis, particularly in microRNA detection. Another well-known NA analog is N-(2-aminoethyl)-glycine-based peptide nucleic acid (PNA) unit, which is a synthetic NA mimic with an achiral and uncharged structure (Figure 4(Ac)). Different bases are linked to this backbone as a side chain by methylene carbonyl groups. As a result, PNA oligonucleotide molecules in uncharged form have high affinity when hybridized with the negatively charged DNA/RNA to form a more stable dsDNA/PNA or RNA/PNA structure in any buffer solution. These hybrid double-stranded NAs have high specificity and accurately matching event, which are beneficial to PNA-based genosensors [59].

#### 3.1.2. Aptasensor

An aptasensor uses the aptamer, an ssDNA sequence with specific recognition ability to one type of chemical target. Similar to the construction of the genosensor, the aptasensor also consists of the specific structure of the ssDNA/RNA ligand that is immobilized on the transducer surface to provide measurable signals for targets that have substantial health indications to humans or animals [61].

Most aptamers are selected using the SELEX method. In principle, the SELEX requires a large library containing more than 10^15^ oligonucleotide sequence structures with a maximum of 60 random synthetic oligonucleotide regions flanked by two constant short regions as primers for PCR amplification [47]. After the target is incubated with oligonucleotide pools under suitable conditions for a while, the unbound aptamers are removed, and the target/aptamers are separated. The separated aptamers are either directly amplified by PCR or amplified by PCR after in vitro transcription and reversed transcription, based on the type of NA (DNA/RNA). The amplified DNA/RNA PCR sequences then enter the subsequent selection round. The final selected aptamer is characterized by its binding kinetics via multiple methods [46]. Depending on the integration methods of SELEX, several SELEX methods have emerged for preferred aptamer selection, including cell-SELEX, microfluidic-SELEX, capillary electrophoresis-SELEX methods, etc. [62,63]. In our group, we obtained the aptamer specific against antibiotic penicillin (PenG) via graphene oxide (MAX)-SELEX method described in Figure 4B [60]. The total, selection rounds are generally divided into (1) positive rounds and (2) negative rounds. During the positive rounds, PenG was incubated with an ssDNA pool under proper conditions where PenG-ssDNA conjugation forms. Subsequently, MAX was introduced to remove unbounded ssDNA, taking advantage of the π–π staking between MAX and free-ssDNA, which are then discarded. The resulting bounded ssDNA were recovered via alcohol to obtain a ssDNA pool for PCR amplification and a subsequent couple of selection processes. The final obtained ssDNA mixture was further repeatedly selected via negative rounds where other interfering antibiotics were introduced to attach and separate less specific ssDNA. Three PenG aptamers were finally obtained (PenG-1, PenG-2, PenG-3), among which PenG-1 showed the lowest dissociation constant (Kd) as 105.15 ± 1.94 nM, indicating that this aptamer has the highest affinity to PenG due to the inverse relationship between the Kd value and binding affinity. Based on the SELEX method, more than a thousand aptamers have been selected to bind different targets, including pathogens, antibiotics, mycotoxins, pollutants, etc., which further extends the applications of aptasensors [64].

#### 3.1.3. NA Enzyme (NAzyme) Biosensor

The notion that all enzymes are proteins has permanently changed since the discovery of RNAs with enzyme function (ribozymes) [65,66]. However, natural ribozymes can only catalyze a limited number of biological reactions, mostly in phosphodiester bond cleavage, splicing, and peptide bond formation [67]. In contrast, artificial ribozymes synthesized and selected by in vitro selection or in vitro evolution have broad catalytic abilities in additional chemical reactions such as phosphodiester bond formation, aminoacylation in coenzyme A for multiple metabolic processes, etc. [68,69]. Later on, artificial DNA molecules were also identified with enzymatic activities (DNAzyme) that allow them to mediate RNA/DNA cleavage and ligation, porphyrin metalation, redox reactions as peroxidases do, etc. [70]. Currently, a new generation of NAs known as aptazymes is being used for biosensing, which integrates aptamer and DNAzyme technologies to specifically identify a target and catalyze biochemical reactions [71].

**Figure 5:**
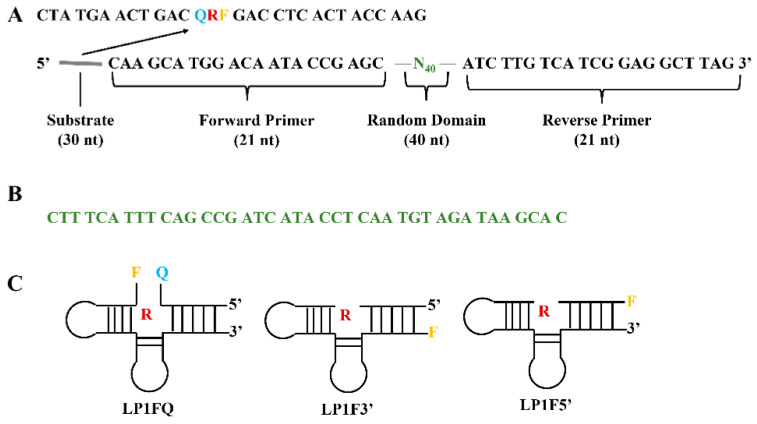
(**A**). The DNA general construct in the library for selection of DNAzyme specific to Legionella pneumphila. (**B**) The random domain of selective Legionella pneumphila DNAzyme. (**C**) Representative construction of selected DNAzyme. R: Adenosine ribonucleotide, F: Fluorescein tail molecules, Q: quencher tail molecules (DABCYL). Redrawn based on Ref. [75].

Once the NAzyme biosensors are assembled for the detection of different targets, the target molecules usually act as cofactors either in accelerating or inhibiting biochemical reactions triggered by the NAzyme probe [72]. Therefore, an appropriate NAzyme biosensor design needs to be considered, as well as the way the catalytic reaction only occurs in the presence of the target. Theoretically, all DNAzyme and aptazyme systems can be employed in biosensors. The most common DNAzyme biosensors are based on the RNA-cleavage DNAzyme probe, taking advantage of both RNA-cleavage DNAzyme and catalytic products released from the RNA-cleavage reaction [73]. The metal ions or amino acids are two critical elements in the RNA-cleavage activity due to the mechanism of the catalytic reaction that requires a specific cofactor during the DNAzyme catalysis. As a result, the preference for using the RNA-cleavage DNAzyme biosensor is for the metal ion analysis or amino acids and peptides detection [74]. An RNA-cleavage fluorogenic DNAzymes (RFDs) for sensing *Legionella pneumphila* was selected and isolated from a DNA pool of 10^14^ oligonucleotide structures containing 40 random nucleotides by utilizing the counter selection method. The structure of the library used for this is shown in Figure 5A [75]. The random domain of RFDs was examined, as shown in Figure 5B. These DNAzymes were tailed with thymidine nucleotide-based fluorophore substrates at the 5′-end and quenchers at the 3′-end (Figure 5C). This unique feature elicits a fluorescence signal in the biosensor. The DNAzymes are highly sensitive to *Legionella pneumophila* strains due to the activation of those bacterial pathogens by specific RNase I protein sequences that were listed as the identity of these strains [76].

### 3.2. Role of MXene in NA-Based Biosensors

The MXene nanoflakes are used in biosensors not only for their large surface area to facilitate NA immobilization for target capture but also their ability to transduce the physiochemical interaction into measurable signals. With the surface chemically terminated by several external single atoms and polar groups, MXene nanosheets are capable of interacting with NA by van der Waals forces, hydrogen bonds, electrostatic attraction, and perhaps coordination bonds, which allows them to be a better material for biosensing system than other 2D NMs [77]. In addition, good biocompatibility and enzyme-responsive biodegradability of MXene nanosheets extend their applications for NA immobilization in the biological area [78,79]. The long-term durability of MXene nanosheets also benefits the reproducibility of biosensor measurements. The merit of MXene nanocomposites is anticipated to be significantly expanded and enhanced as they incorporate other nano(bio)materials. Kashefi-Kheyrabadi et al. [80] designed an electrochemical aptasensor for thyroxine analysis by immobilizing thyroxine aptamer on the MoS_2_/Ti_3_C_2_T_x_ MXene/Au nanosheets, with a limit of detection (LOD) of 0.39 pg/mL and a dynamic range from 7.8 × 10^−1^ to 7.8 × 10^6^ pg/mL.

## 4. Application of MXene-Based NA Biosensors in the Agricultural Food System

As an emerging class of 2D NMs, MXene nanosheets have proven to be suitable transducer materials for biosensor applications. Hence, various NA biosensors with MXene as transducer NM have been developed for quality and safety monitoring of food products as listed in Table 1.

### 4.1. Pathogen Detection

The presence of pathogenic microorganisms in agri-food systems is a growing global concern for human and animal health. If not promptly detected, the spread of these pathogens through the food supply chain can become a major problem causing illnesses and loss of lives. Hence, simple and rapid biosensors are being developed for the detection of pathogens.

Wang et al. [82] developed an electrochemical and colorimetric signal-producing biosensor for the on-site detection of *Vibrio parahaemolyticus* (VP). VP is a gram-negative bacteria found in the ocean and estuaries that can cause inflammatory gastroenteritis in humans [112]. The VP detection system was developed using phenylboronic acid and ferrocene-modified platinum (Pt)-doped Ti_3_C_2_ MXene nanocomposites (PBA-Fc@Pt@MXene). The use of PBA-Fc@Pt@MXene provided high electrical conductivity and significantly enhanced the electrochemical signal intensity and produced a color change when the existence of hydrogen peroxide (H_2_O_2_) and 3,3′,5,5′-tetramethylbenzidine (TMB) was detected. Incorporated with a VP aptamer, this biosensor can both voltammetrically determine and colorimetrically visualize the presence of VP as low as 5 CFU/mL and 30 CFU/mL, respectively. The linear range for electrochemical measurement and colorimetric determination is 10–10^8^ CFU/mL and 10^2^–10^8^ CFU/mL, respectively. This fabricated biosensing device provides a dual-mode detection method for highly effective VP detection and measurement.

**Figure 6:**
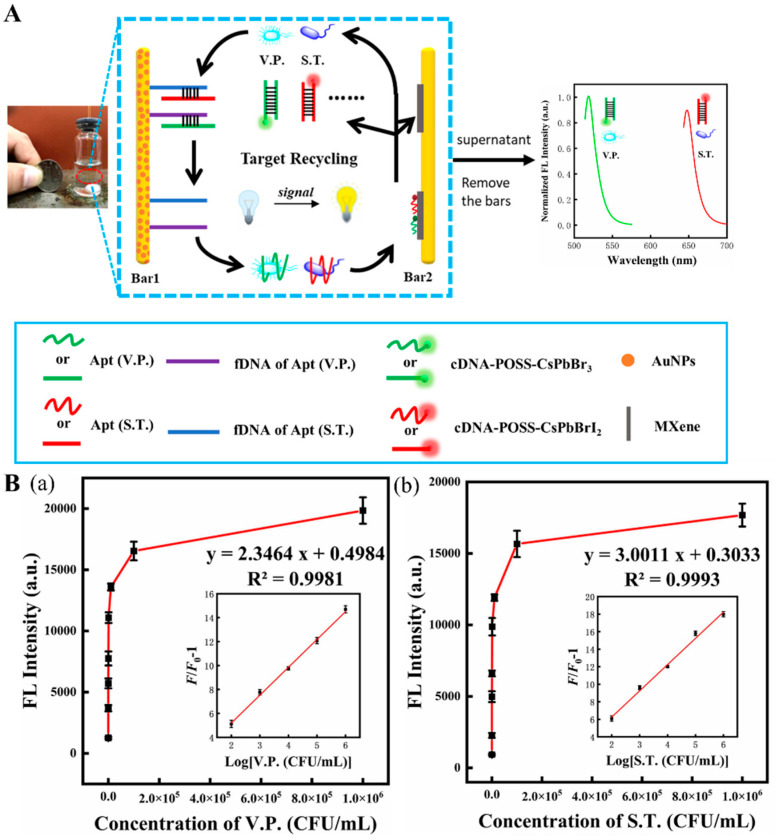
(**A**) Scheme of fluorescent aptasensor for dual-targets detection with its signal amplification strategy. (**B**) (**a**,**b**) the fluorescence intensity of the aptasensor in the presence of VP (**a**) and ST (**b**). Inset: linear range for this aptasensor for two corresponding foodborne pathogens. Reproduced with permission from Ref. [81].

*Salmonella typhimurium* (ST) is another dangerous foodborne pathogen that causes high morbidity and mortality [113]. A novel dual-target fluorescent aptasensor was fabricated for live ST and VP analysis [81]. As shown in Figure 6A, this fluorescent device was assembled by two magnetic bars (1 and 2), where bar 1 was used as a biorecognizer for target recognition and capture, and bar 2 served as a signal amplifier that released the enhanced fluorescent signal. Bar 1 was coated with aptamers capable of recognizing and capturing two bacteria strains. Bar 2, modified with Ti_3_C_2_ MXene nanosheets, served as a platform to support and immobilize complementary DNA (cDNA) tailed with polyhedral oligomeric silsesquioxane-perovskite quantum dots probes (cDNA-POSS-PQDs). When the two target bacteria were present, they were captured by their specific aptamers on bar 1 and subsequently released with their aptamers into the aqueous supernatant. Due to the agitation, bacteria–aptamer conjugate attached to the cDNA on bar 2 and then tore away the cDNA-POSS-PQDs from bar 2, which finally provided a fluorescent response in the solution. The live bacteria can be released from the aptamer and become free again due to the reaction between the aptamer and cDNA-POSS-PQD with low Gibbs free energy (∆G < 0), leading them to trigger the next detection cycle. Ultimately, the fluorescent response was amplified and reached a maximum that significantly increased the biosensor sensitivity. LODs of 30 CFU/mL and 10 CFU/mL, respectively, for ST and VP, and a linear range of 10^2^ to 10^6^ CFU/mL were obtained (Figure 6(Ba,Bb)). Therefore, this biosensor can sensitively detect the target bacteria and differentiate the live from the dead ones.

Simply using MXene and MXene-based nanocomposites for the NA immobilization yields only weak, unstable signals due to the activity of the live pathogens which may (1) escape or detach from the aptamer capture and stay free in the solution, and (2) disturb the signal when they are struggling with the aptamer capture. Hence, a MXene-based signal amplifier is used in NA biosensors to facilitate the on-site determination of live pathogens.

### 4.2. Mycotoxins Detection

Mycotoxins are toxic secondary fungal metabolites found in plant and animal foods. The consumption of some mycotoxin-containing foods beyond certain recommended amounts can cause serious health issues, including death. A sensor for the detection of mycotoxins in food and feed products will help enable a strategy to mitigate adverse health effects in humans and animals.

**Figure 7:**
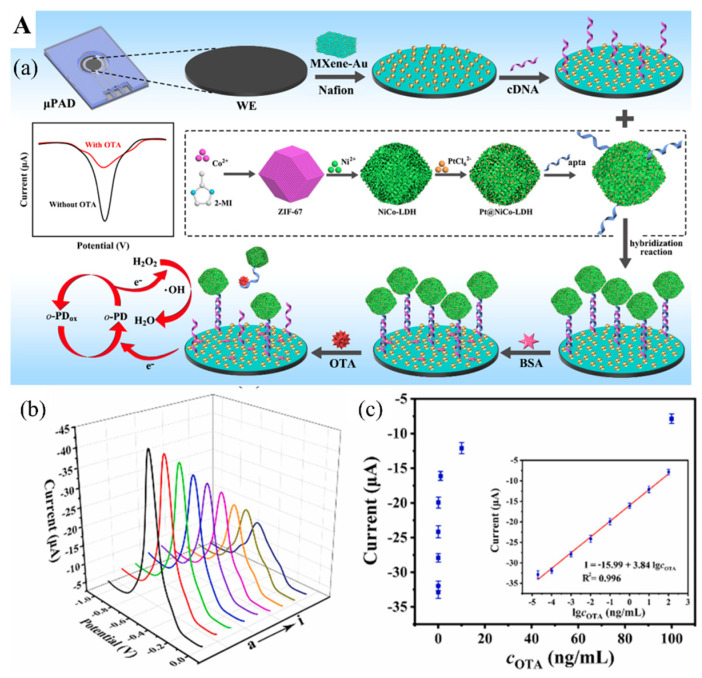

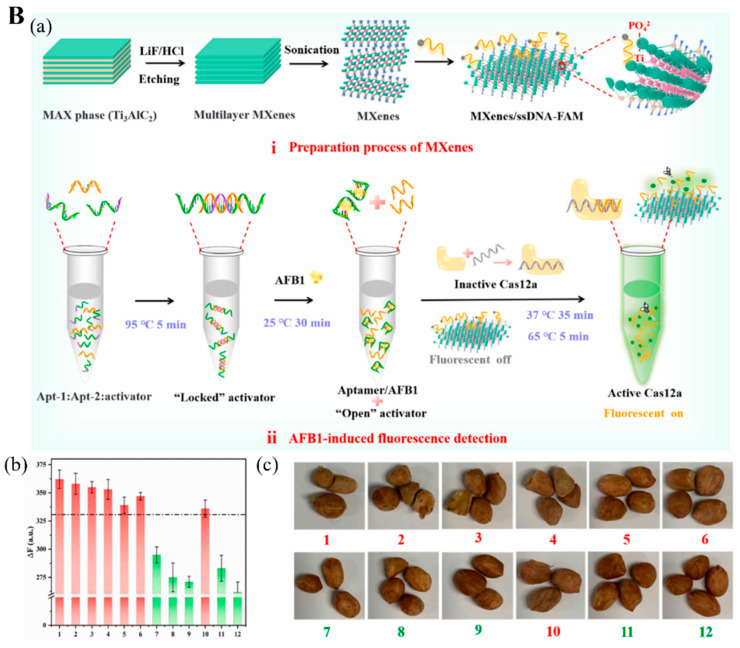
(**A**) (**a**) Diagram of the assembly process of electrochemical aptasensor for OTA analysis. (**b**) Voltammetric response of aptasensor in the presence of different OTA concentrations. (**c**) Relationship between the electrochemical response and OTA concentration. Inset. The linear relationship between the response and OTA concentration. Reproduced with permission from Ref. [87]. Copyright 2022, Elsevier. (**B**) (**a**) The construction of CRISPR/Cas12a-based fluorescent biosensor for AFB1 determination. (**b**) Measurement of AFB1 level in 12 peanut samples by the constructed fluorescent biosensor. (**c**) Pictures of 12 peanut samples tested positive (red) and negative (green). Reproduced from Ref. [92].

Zhang et al. [87] developed a switchable signal electrochemical aptasensor for the detection of mycotoxin Ochratoxin A (OTA) via a substrate-aptamer-signal amplifier sandwich structure. The OTA aptamer cDNA with a thiol group (-SH) was immobilized on the surface of MXene/Au nanocomposites-coated paper device via Au-S covalent bond (Figure 7(Aa)), employed as the transducer substrate coated on the working electrode. OTA aptamers tailed with peroxidase mimic nanostructure based on Pt nanoparticles-doped NiCo hollow layer double hydroxides (Pt@NiCo-LDH) were adopted to hybridize with their cDNA to form a rigid dsDNA helix, which kept the “signal-on” state (Figure 7(Aa)). As the OTA-contaminated sample is tested, OTA aptamers conjugating with OTA on their active sites result in the detachment of OTA aptamers from their cDNA. Without the nanostructure amplifier, this NA biosensor significantly decreased its electrochemical performance and switched to a “signal-off” state, thus lowering the electrical signals. This biosensor can detect OTA concentration as low as 8.9 fg/mL in aqueous samples (Figure 7(Ab,Ac)). A surface-enhanced Raman scattering aptasensor has also been assembled for OTA measurement based on the Ti_3_C_2_O_2_ MXene. The Raman signal was enhanced by plasmonic OTA aptamers modified with Au-Ag Janus nanocomposites [88]. In the absence of OTA, the OTA aptamer/Au–Ag conjugates bind to the MXene surface by hydrogen bond and chelation interaction between the phosphate group of ssDNA and Ti ions, providing an amplified Raman signal. However, in the presence of OTA, the OTA aptamer/Au–Ag conjugates dissociate from the MXene surface since the active sites of ssDNA are blocked by OTA. The Raman signal thus was attenuated to MXene internal signal without Au–Ag Janus nanocomposites. Consequently, an ultralow LOD of 1.28 pM was achieved and was used to test red wine samples for OTA. The presence of OTA was also detected by a DNAzyme-based biosensor [89]. The Ti_3_C_2_ MXene-TiO_2_ nanocomposites were doped with Au@PtAg nanoparticles that were a part of the platform for facilitating the photogenerated electron transfer, supporting the ferrocene-labeled duplex DNA probe. With DNAzyme cascade amplification, this photoelectrochemical DNAzyme-based biosensor can rapidly measure the concentration of OTA from 5 fg/mL to 10 ng/mL with a LOD of 1.73 fg/mL. Therefore, this device might be more effective for on-site detection of OTA.

The clustered regularly interspaced short palindromic repeats and specific proteins (CRISPR-Cas) technique was also used for detecting aflatoxin B1 (AFB1) and deoxynivalenol (DON) [92,94]. AFB1 is a secondary toxic metabolite released from *Aspergillus flavus* and *A. parasiticus,* and *A. nominus*, frequently found in various foods, including peanuts, grains, and animal feed [114]. Wu et al. [92] explored a MXene-based fluorescence biosensor incorporated with the CRISPER-Cas12 technique for AFB1 measurement. When AFB1 is present in the sample, it conjugates with one ssDNA, a locked activator in the form of helical double-strand aptamer opens and releases an aptamer to activate the inactive Cas12a protein linked with guide RNA (crRNA) and thus cleaves the quenched fluorophore-modified ssDNA immobilized on the MXene as small fragments (Figure 7(Ba)). These fragments then leave the MXene and are dispersed into the solution, recovering the fluorescence signal to the aqueous environment. Their tests in 12 peanut samples for AFB1 were highly accurate (Figure 7(Bb,Bc)).

Lin et al. [94] designed a luminescent aptasensor for the detection of DON using an aptamer to activate Cas12 protein trans-cleavage activity that facilitated the luminophore released from the MXene-Au platform but was suppressed in the presence of DON, which finally produced an on/off signal.

Other mycotoxins, such as gliotoxin containing di- or polysulfide bridges, can induce cytotoxicity in humans and animals [115]. A tetrahedral DNA nanostructure (TDN) was grown on the surface of Ti_3_C_2_ MXene with one extended capture aptamer in a stand-up posture from a vertex. Combined with the merit of signal enhancers, this MXene-based TDN biosensor can measure gliotoxin in human serum samples as low as 5 pM [96].

### 4.3. Antibiotics Detection

The presence of antibiotics and antibiotic-based medicines is also among the public concerns regarding food safety. The use of antibiotics and related drugs in agri-food systems is often necessary to curb bacterial invasion. Nevertheless, excessive or improper usage of antibiotics leads to food and feed sources laced with antibiotic residues. Consumption of such food and feed products can not only cause several adverse side effects in humans and animals, but also enhance antibacterial resistance [116,117].

Chloramphenicol (CAP) is one of the common antibiotics banned from food production due to its potential for bone marrow aplasia, gray-baby syndrome, etc., but it still probably exists in foods of animal origin [118]. Yang et al. [100] developed an electrochemical aptasensor using CAP aptamer immobilized on the MXene surface for the detection of CAP in honey as low as 0.03 pM. In another effort, Jiang et al. [101] employed ZnO quantum dots/nitrogen (N)-doped MXene as a sensing framework that supported aptamers to interact with CAP specifically. Their electrochemiluminescence aptasensor could detect CAP in water and milk with a LOD of 0.019 ng/mL and a linear range from 0.1 to 100 ng/mL.

**Figure 8:**
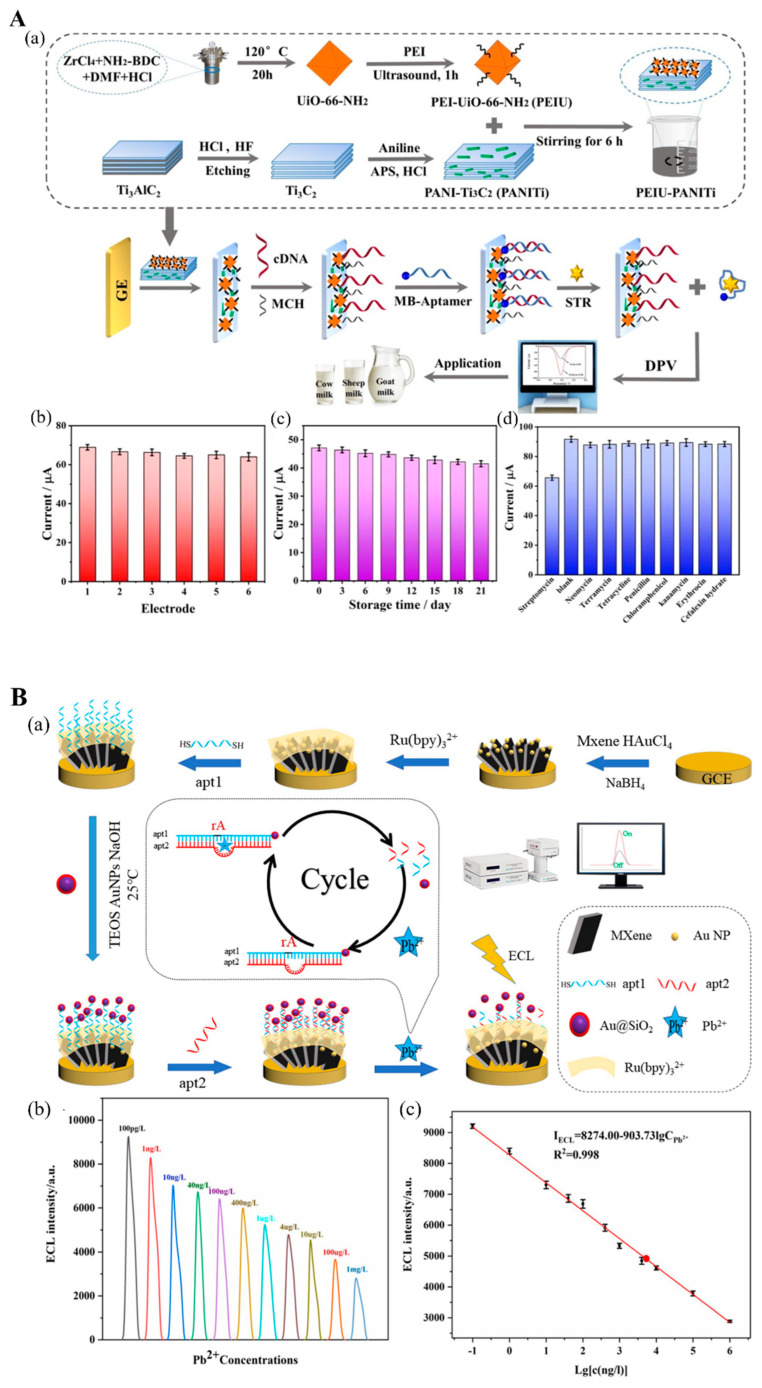
(**A**) (**a**) Illustration of STR electrochemical aptasensor construction. (**b**) Electrochemical response for six replications of STR detection. (**c**) Aptasensor stability after storing for 21 days. (**d**) Response of electrochemical signal for STR and interference evaluation. Reproduced with permission from Ref. [103]. Copyright 2022, Elsevier. (**B**) (**a**) Illustration of electrochemiluminescent (ECL) DNAzyme-based biosensor assembly for Pb^2+^ detection. (**b**) Responses of ECL of assembled DNAzyme-based biosensor in the presence of various Pb^2+^ concentrations. (**c**) The linear range of ECL biosensors for Pb^2+^ measurement. Reproduced with permission from Ref. [107].

Another important antibiotic family is aminoglycosides, which are natural or semisynthetic actinomycetes derivatives, including streptomycin (STR), kanamycin, neomycin, tobramycin, etc. [119]. You et al. [102] devised a photoelectrochemical “on-off-on” aptasensor for STR measurement with STR aptamer-modified Bi_4_VO_8_Br/Ti_3_C_2_ nanostructure. The ability of the Bi_4_VO_8_Br/Ti_3_C_2_ nanostructure to generate photogenerated signals is efficiently inhibited by the STR aptamer, resulting in an “on-off” signal framework. When STR is present, the STR aptamer–STR conjugate lowers the inhibition ability of STR. Hence, the photoelectrochemical signal from Bi_4_VO_8_Br/Ti_3_C_2_ is again generated. This “on-off-on” aptasensing scheme was able to measure STR concentration in honey in the range of 1 to 1000 nM. In another electrochemical aptasensor, polyethyleneimine (PEI) was used in a metal–organic framework (PEI-UIO-66-NH_3_) to coat the polyaniline-Ti_3_C_2_ MXene surface (PANI-MXene), which was used to modify a glassy electrode (Figure 8(Aa)) [103]. After incubating with cDNA, the electrode was ready to immobilize the STR aptamer-methylene blue (MB) structure, which is released from the electrode in the presence of STR. This aptasensor successfully detected 0.01 to 200 nM STR in milk (Figure 8(Ab–Ad)).

MXene-based NA biosensors have also been developed for the detection of enrofloxacin and ciprofloxacin [104,105]. Enrofloxacin and ciprofloxacin are synthetic fluoroquinolone antibiotics and are used in veterinary drugs. Jiang et al. [104] fabricated an electrochemiluminescence aptasensor based on O-terminated Ti_3_C_2_ MXene doped with AgBr nanocrystals. The LOD of this sensor for enrofloxacin in pond water was 5.97 × 10^−13^ mol/L. The aptamer/Ti_3_C_2_-Bi_4_VO_8_Br-TiO_2_ nano-construction was used for photoelectrochemical detection of ciprofloxacin, which provides the effectiveness of health implications to milk samples [105].

### 4.4. Other Targets

MXene-based NA biosensors have also been developed for the detection of other target analytes in the agri-food systems, such as the presence of heavy metals. Metal ions are essential to life. However, overconsumption of heavy metal-containing food and feed products causes health issues, e.g., cancer, renal damage, nervous system disruption, etc. Mercury (Hg^2+^) and lead (Pb^2+^) poisoning are responsible for several diseases and even death due to the ingestion of contaminated food or drinks [1]. In an electrochemical biosensor designed by Liu et al. [106], Ti_3_C_2_T_x_ MXene/Nafion was modified by GR5 DNAzyme that can cleave the ribo-adenine (rA) site of the DNA substrate in the presence of Pb^2+^. Here, Nafion with high viscosity can function as an adhesive not only for Ti_3_C_2_T_x_ MXene-GR5 DNAzyme binding but also for Ti_3_C_2_T_x_ MXene immobilization over the sensing substrate. After DNA substrate cleavage, the absorptivity of MXene is largely improved, facilitating the adoption of GR5 DNAzyme and ion intercalation, thus increasing its electrochemical performance. This DNAzyme-based biosensor can detect the Pb^2+^ effectively and selectively for food safety and environment monitoring. Zhai et al.[107] employed an electrochemiluminescent biosensor for Pb^2+^ in four water samples. They used MXene@Au loaded with tris(2,2-bipyridyl) ruthenium (II) (Ru(bpy)_3_^2+^) as a transducer platform to support the aptazyme and its rA-containing DNA substrate labeled with Au@SiO_2_ luminophore (Figure 8(Ba)). Pb^2+^ ions activated the endonuclease behavior of the aptazyme that cut the rA sites of the DNA substrate, leading to the luminophore-tailed sequence of DNA substrate releasing into the solution, thus reducing the electrochemiluminescence signal generated from the biosensor. Based on this, they achieved an LOD of 0.059 ng/L over the detection rage of 0.1 to 10^6^ ng/L for Pb^2+^ (Figure 8(Bb,Bc)).

A DNAzyme-based fluorometric biosensor was proposed by Lu et al. [110] for the detection of Hg^2+^. Principally, Ti_3_C_2_ MXene nanosheets adsorbed the fluorophore-labeled ssDNA by metal chelation interaction and hydrogen bond but quenching the fluorescence effect of a fluorophore. The introduction of H^2+^ ions initiated the hybridization reaction between the exposed sequence of hairpin DNA and free primers (hairpin-Hg^2+^-primer) that were further digested by exonuclease III. Then, the hairpin-Hg^2+^-primer structure was broken down into several small ssDNA sequences and nickers that hybridized with fluorophore-tailed ssDNA, thus facilitating the fluorophore-tailed ssDNA separated from the MXene surface to generate a fluorescent signal. This sensor was capable of detecting Hg^2+^ in water as low as 42.5 pM.

Other deleterious contaminants in the agri-food systems include pesticide residues. As an organophosphorus insecticide and acaricide, isocarbophos (ICP) can induce several acute toxicities and are carcinogenic to humans. Zhi et al. [111] developed a Ti_3_C_2_ MXene@Au sol support ICP aptamer probe using the catalytic activity of the sol to reduce mandelic acid-chloroauric acid (MA-HAuCl_4_) for a dual-mode measurement of the ICP concentration in farmland wastewater with high stability, reproducibility, and specificity.

## 5. Summary and Future Perspectives

MXene-based NA biosensors offer high sensitivity that can help quantitively measure the presence of various analytes down to picomolar concentrations. This has prompted the development of various biosensors for different applications, enabling the implementation of preventive strategies at the source of the agri-food systems.

MXenes are usually employed as transducer elements due to their unique physical and electrical properties. However, the effectiveness of signal transmission may vary depending on the quality of MXene. In addition, the short life of NA impedes the development of robust MXene-based NA biosensors. Furthermore, the oxidation of MXene may impair the long-term functionality and stability of MXene biosensors, especially those that are employed in hot and humid environments, which is often the case for on-site testing of agri-food products. The cost of the MAX phase, the MXene precursor, is rather high compared to other 2D NM precursors such as graphite. In addition to the above challenges, the biggest challenge currently faced is the real-world application of MXene-NA biosensors, e.g., in food or environmental sample matrices such as corn or wheat flour, fruit juice, milk, or soil where a lot of interfering miro/nanoparticles may generate noise signals when they nonspecifically attach to the biosensor.

To improve the performance of NA biosensors based on MXene and address the challenges mentioned above, we offer the following. First, it is essential to develop a complete understanding of how MXene and NA interact. Though several investigations have suggested that certain ssDNA can chemically conjugate with MXene, there is still a lack of definitive confirmation of this. Second, there is a need to develop highly stable ssDNA mimics that have specific recognition abilities. Third, since in agri-food systems, on-site testing is a high priority, portable MXene-based NA biosensors such as lateral flow assays and lab-on-a-chip devices are needed. Some MXenes other than Ti_3_C_2_T_x_ such as Nb_4_C_3_ may be a suitable substitute that can be widely adopted in the NA biosensors for chemical target detection.

In summary, despite these recent developments, the technology of MXene-based NA biosensors is still in its relative infancy. We expect many exciting developments in the next five to ten years, responding to the need for on-site testing of complex real matrices in agri-food systems.

## Figures and Tables

**Table 1:** Summary of MXene-based NA biosensors for agri-food systems.

Analytes		MXene Biosensor	Detection Methods	LOD	Linear Range	Real Sample	Refs.
Foodborne pathogens	*Salmonella typhimurium*	Aptamer/cDNA- PQDs/Ti_3_C_2_ MXene nanosheets	Fluorescent	30 CFU/mL	10^2^–10^6^ CFU/mL	Real aquatic sample	[81]
	*Vibrio parahaemolyticus*			10 CFU/mL			
	*Vibrio parahaemolyticus*	Aptamers/PBA-Fc@Pt@MXene	Electrochemical	5 CFU/mL	10–10^8^ CFU/mL	Shrimp and water	[82]
			Colorimetric	30 CFU/mL	10^2^–10^8^ CFU/mL		
		MXene/POSS-PQD-Apt	Fluorescent	30 CFU/mL	10^2^–10^6^ CFU/mL	Seawater	[83]
	*Mycobacterium tuberculosis*	PNA/Zr-MXene	Electrochemical	20 CFU/mL	10^2^–10^8^ CFU/mL	Simulated sputum sample	[84]
		Ti_3_C_2_ MXene/polypyrrole/methylene blue	Electrochemical	11.24 fM	100 fM–25 nM	Clinical human sputum	[85]
	*Escherichia coli*	MXene/CRSPR-Cas12a/ssDNA-fluorophore	Fluorescent	23 CFU/mL	3.2 × 10–3.2 × 10^7^ CFU/mL	Purified water, milk, grapefruit juice, green tea	[86]
	Lipopolysaccharide			11 pg/mL	0.1–8000 ng/mL		
Mycotoxin	Ochratoxin A	Aptamer/cDNA-MXene-Au/Pt@NiCo-LDH	Electrochemical	8.9 fg/mL	20 fg/mL–100 ng/mL	Corn and wheat sample	[87]
		MXene/Aptamer/Au-Ag Janus nanocomposites	Surface-Enhanced Raman Scattering	1.28 pM	0.01–50 nM	Red wine sample	[88]
		DNAzyme cascade amplification/MXene-TiO_2_/Au@PtAg	Photoelectrochemical	1.73 fg/mL	5 fg/mL–10 ng/mL	-	[89]
		Au@ MXene/tetrahedral DNA/signal probe DNA-Au@MOF (UIO-66)	Electrochemical	330 fg/mL	1 pg/mL–100 ng/mL	Corn sample	[90]
		MXene/polyvinylidene fluoride nanofiber/ Aptamer	Electrochemical	2.5 fg/mL	1 fg/mL–1 ng/mL	Grape juice	[91]
	Aflatoxin B1	MXene/fluorophore-modified ssDNA/ Aptamer-CRISPR-Cas12	Fluorescent	0.92 pg/mL	0.001–80 ng/mL	Peanut sample	[92]
		MXene/Au dimer-Ag/ aptamer	Surface-Enhanced Raman Scattering	0.6 pg/mL	0.001–100 ng/mL	Peanut sample	[93]
	Deoxynivalenol	MXene-Au/luminophore-modified ssDNA/ Aptamer-CRISPR-Cas12	Luminescent	0.64 ng/mL	1–500 ng/mL	Corn and lake water	[94]
		MXene/Aptamer/APTES-glutaraldehyde	Electrochemical	1 fg/mL	1 fg/mL–1 ng/mL	Paddy plant extractions	[95]
	Gliotoxin	MXene/tetrahedral DNA nanostructure/cDNA-peroxidase polymer	Electrochemical	5 pM	5 pM–10 nM	Human serum sample	[96]
	Zearalenone	MXene/Chitosan/Aptamer/APTES-glutaraldehyde	Electrochemical	0.4 pg/mL	1 fg/mL–1 ng/mL	Corn and cow milk	[97]
	Microcystin-LR	MXene@Au nanocomposites/cDNA-methylene blue/reduced graphene oxide/Au/Aptamer	Electrochemical	4 × 10^−5^ nM	0.0001–5 nM	Tap water and surface water	[98]
	Saxitoxin	MXene/silane coupling agent/Aptamer	Electrochemical	0.03 nM	1.0–200 nM	Mussel tissue	[99]
Antibiotics	Chloramphenicol	Aptamer/MXene	Electrochemical	0.03 pM	0.0001–10 nM	Honey sample	[100]
		Aptamer/ZnO quantum dots-N doped MXene	Electrochemiluminescence	0.019 ng/mL	0.1–100 ng/mL	Pond water and milk	[101]
	Streptomycin	Aptamer/Bi_4_VO_8_Br/Ti_3_C_2_ nanostructure	Photoelectrochemical	0.3 nM	1–1000 nM	Honey sample	[102]
		cDNA/Aptamer/MOF (UIO-66-NH_2_-PEI)/PANI-Ti_3_C_2_ nanostructure	Electrochemical	0.0033 nM	0.01–200 nM	Milk samples	[103]
	Enrofloxacin	Aptamer/O-Ti_3_C_2_ MXene-AgBr nanocrystal	Electrochemiluminescence	5.97 × 10^−13^ mol/L	1.0 × 10^−12^–1.0 × 10^−6^ mol/L	Pond water and tap water	[104]
	Ciprofloxacin	Aptamer/Ti_3_C_2_-Bi_4_VO_8_Br-TiO_2_	Photoelectrochemical	0.3 nM	1–1500 nM	Milk samples	[105]
Metal ions	Pb^2+^	Nafion/Ti_3_C_2_T_x_ MXene/GR5 DNAzyme	Electrochemical	0.1 nM	0.5–32 nM	Liver tissues of rat and chicken	[106]
		Aptazyme/cDNA/Au@SiO_2_- Ru(bpy)_3_^2+^/Ti_3_C_2_T_x_ MXene@Au	Electrochemiluminescence	0.059 ng/L	0.1 –10^6^ ng/L	Tap, lake and industrial waste water	[107]
		MXene/FAM-DNA substrate/GR5 DNAzyme	Fluorescent	0.05 ng/mL	0.2–10 ng/L	Tap and river water	[108]
		Au@Nb_4_C_3_T_x_ MXene/ Aptamer	Electrochemical	4 nM	10 nM–5 µM	Tap and bottled water	[109]
	Hg^2+^	Aptazyme/fluorophore-cDNA/Exo III-assisted system Ti_3_C_2_T_x_ MXene	Fluorescent	42.5 pM	0.05–50 nM	Tap and river water	[110]
Insecticides	- Isocarbophos (ICP)	MXene@Au/ICP aptamer/mandelic acid-HAuCl _4_	Surface-Enhanced Raman Scattering	4.5 × 10^−5^ nmol/L	1.0 × 10^−3^–2.5 × 10^−2^ nmol/L	Farm water	[111]

## Data Availability

Not applicable.
